# Global DNA Methylation Patterns in Human Gliomas and Their Interplay with Other Epigenetic Modifications

**DOI:** 10.3390/ijms20143478

**Published:** 2019-07-15

**Authors:** Michal J. Dabrowski, Bartosz Wojtas

**Affiliations:** 1 Institute of Computer Science, Polish Academy of Sciences, 01-248 Warsaw, Poland; 2 Nencki Institute of Experimental Biology, 02-093 Warsaw, Poland

**Keywords:** chromatin, CpG, cytosine, DNA methylation, G-CIMP, glioblastoma, glioma, histone, IDH

## Abstract

During the last two decades, several international consortia have been established to unveil the molecular background of human cancers including gliomas. As a result, a huge outbreak of new genetic and epigenetic data appeared. It was not only shown that gliomas share some specific DNA sequence aberrations, but they also present common alterations of chromatin. Many researchers have reported specific epigenetic features, such as DNA methylation and histone modifications being involved in tumor pathobiology. Unlike mutations in DNA, epigenetic changes are more global in nature. Moreover, many studies have shown an interplay between different types of epigenetic changes. Alterations in DNA methylation in gliomas are one of the best described epigenetic changes underlying human pathology. In the following work, we present the state of knowledge about global DNA methylation patterns in gliomas and their interplay with histone modifications that may affect transcription factor binding, global gene expression and chromatin conformation. Apart from summarizing the impact of global DNA methylation on glioma pathobiology, we provide an extract of key mechanisms of DNA methylation machinery.

## 1. Methylation and Demethylation

DNA methylation is a covalent transfer of a methyl group to a DNA base and occurs either as a result of action of alkylating DNA damaging agents or is a precisely controlled event leading to epigenetic modification of DNA. Damage-related methylation involves generation of N1-methyladenine (m1A) or N3-methylcytosine (m3C), both of which are introduced by endogenous or exogenous methylating agents. Such changes are described as cytotoxic or mutagenic because they are able to block or alter Watson–Crick base-pairing [1]. Another category of DNA methylation is associated with a specific modification (mainly at position 5 of cytosine, 5mC), that is recognized as the carrier of epigenetic information and influences many regulatory functions in cell development [2]. The latter type of DNA methylation is in the scope of the current review.

In 1975 methylation of cytosine was suggested to play a significant role as an epigenetic mark in animals [3,4]. Currently, DNA methylation is probably the best-studied covalent epigenetic modification. DNA methylation is chemically stable, which makes it one of the most reliable ways to transmit epigenetic information during cells propagation in order to keep the appropriate state of gene expression [2]. In mammalian genomes, DNA methylation most often occurs at the five position (C5) of cytosine, generating 5-methylcytosine (5mC), usually in the CpG dinucleotides context (C = cytosine, p = phosphate bond and G = guanine). DNA methylation in plants or fungi more frequently occurs in the non CpG-context, that is at C bases upstream to DNA nucleotides other than G [5,6]. The non-CpG DNA methylations were thought to be present only in plants and in mammalian embryonic stem cells (ESCs) and then to be lost during the cell differentiation process [7]. Recently, more evidence has been gathered to confirm the presence of non-CpG methylation in mammalian stem cells. Although the presence of the non-CpG methylation in the human fetal brain is negligible, it is abundant in human adult brain neuronal cells [8,9], as well as in other types of differentiated mammalian cells from over a dozen of human tissues [10]. Non-CpG methylations were not only found to be present but also functional in many types of cells [10,11,12].

**Figure 1:**
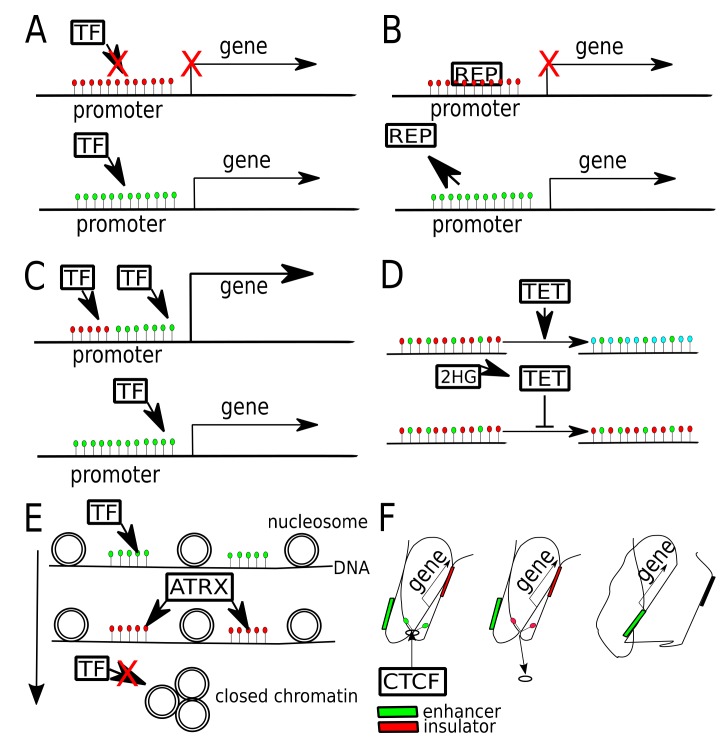
Different ways that DNA methylation may modulate gene expression in gliomas. (**A**) Gene promoter hypermethylation (red dots—5mC) may cause gene expression silencing, by blocking transcription factor (TF) binding. Once promoter gets unmethylated (green dots—unmethylated C), gene expression may occur. (**B**) Gene promoter, when hypermethylated binds transcriptional repressor (REP). Once promoter gets unmethylated, repressor is released, allowing gene expression. (**C**) Binding of two TFs to one promoter boosts gene expression, one of TFs binds preferentially to methylated, while the other to unmethylated DNA. (**D**) In physiological conditions TET proteins oxidize 5mC (red dots) to 5hmC (blue dots) and later to other derivatives (5fC, 5caC). When TET gets inhibited by 2HG (2-hydroxyglutarate), produced by mutant *IDH1/2*, it fails to demethylate DNA, thus maintaining global DNA hypermethylation. (**E**) DNA methylation is related with chromatin openness, due to chromatin chaperons, that are sensitive to DNA and histone being activated or repressed. ATRX protein may bind to methylated DNA and lead to heterochromatin formation, blocking access of TFs. (**F**) Hypermethylation of CTCF binding site causes CTCF unbinding and change of chromatin conformation, which leads to exchange of insulator by enhancer in a close proximity of a gene.

The genomic regions with high frequency of CpGs are referred to as CpG islands. CpG islands are generally hypomethylated in normal cells [13], while being hypermethylated in cancer cells [14]. The methylation level of CpG islands in gene promoters is typically associated with the repression of transcription (Figure 1A,B) [2]. However, in more recent studies several exceptions of that general pattern have been found. For example, high levels of CpG island methylation in the *FOXA2* gene promoter region result in the activation of gene expression. CpG island methylation, which increases during differentiation in endoderm lineage, causes a loss of repressor protein binding and leads to upregulation of *FOXA2* expression [15].

The addition of methyl groups to cytosine residues is mediated in mammals by proteins belonging to the DNA methyltransferases (DNMTs) comprising three proteins assigned to two families, which are structurally and functionally different. DNMTs are highly conserved across various taxa including plants and mammals [5]. In many genomes, starting from bacteria through plants to mammals, genes encoding DNMTs were confirmed to be present, although DNA methylation varies significantly across species [5,16]. The two DNMTs families are defined according to their role in *de novo* methylation or in the maintenance of methylation marks [2]. Generally, initial DNA methylation patterns are established by the *de novo* DNA methyltransferases, such as DNMT3A and DNMT3B. Afterwards, during DNA replication and cell division the initial methylation marks are maintained in daughter cells by the maintenance methyltransferase DNMT1.

The maintenance methyltransferase DNMT1 preferentially methylates hemimethylated DNA, i.e., DNA where only one strand, of the two complementary strands, is methylated (5mC). Methylation of hemimethylated substrate is a highly processive reaction, which means that an enzyme transfers more than one methyl group to one DNA molecule without releasing it. After the transfer of a methyl group there is almost 99% probability that DNMT1 will continue DNA methylation of the same DNA molecule [17]. Due to that the higher methylation rate of DNMT1 on longer DNA molecules was shown [18]. The methylation reaction had significantly reduced rate when CG sites were methylated or unmethylated on both complementary DNA strands in the substrate molecule [17]. It was documented that methylation of hemimethylated CpG sites by DNMT1 at DNA replication forks needs a presence of the cofactor UHRF1 (E3 ubiquitin-protein ligase also known as NP95).

**Figure 2:**
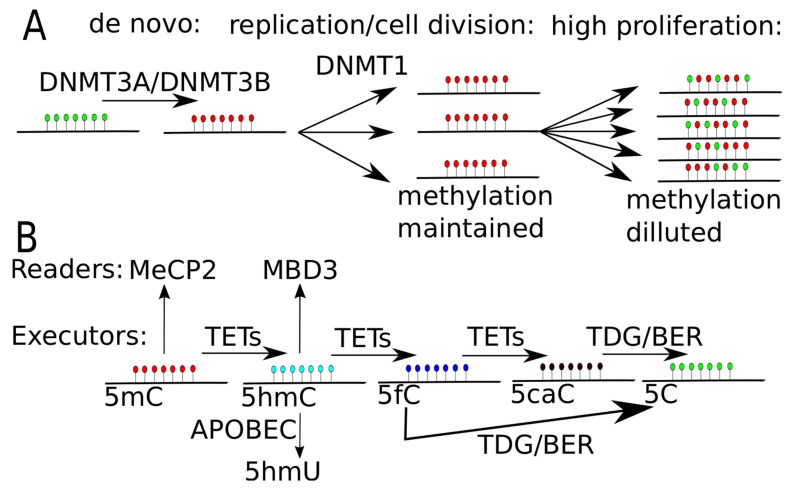
DNA methylation and demethylation. (**A**) DNA methylation through DNA methyltransferase (DNMT) proteins. DNMT3A and DNMT3B put *de novo* methylation, while DNMT1 maintain DNA methylation level during cell replication and cell division. In highly proliferating cells or in cells with decreased level of activity of DNMT1, the signal of DNA methylation gets diluted, leading to passive DNA demethylation. (**B**) DNA demethylation through TET proteins. TET proteins oxidize methylated cytosine (5mC) to hydroxyl-methylated (5hmC), then to 5-formylcytosine (5fC) and finally to 5-carboxylcytosine (5caC). 5fC or 5caC is recognized and excised by thymine-DNA glycosylase (TDG) and replaced by unmodified cytosine by base excision repair mechanism (BER). 5mC can be specifically recognized by MeCP2 (methyl-CpG binding protein 2) and 5hmC may be recognized by MBD3 (methyl-CpG-binding domain protein 3). 5hmC may be transformed to 5hmU by APOBEC (apolipoprotein B mRNA editing enzyme, catalytic polypeptide-like) system, leading to single nucleotide substitution, as uracil in process of replication is soon changed into thymine.

The other group, *de novo* DNMTs, contains DNMT3 family members named DNMT3A and DNMT3B and one regulatory factor DNMT3-like protein (DNMT3L), which is catalytically inactive. In general, DNMT3A and DNMT3B are closely related due to similar arrangements of the protein domains. Both of them have almost identical methyl transfer activity on hemimethylated and unmethylated CpG-contacting sites [19,20]. The N-terminal part of DNMT3A specifically recognizes the histone 3 lysine 36 trimethylation mark (H3K36me3). The interaction of DNMT3A domain with H3K36me3 increases methylation activity of DNMT3A resulting in DNA methylation [21]. DNMT3A and DNMT3B establish an initial CpG methylation pattern *de novo* during the blastocyst stage of embryonic development [19,22,23]. A schematic view on DNMT function is shown in Figure 2A.

Almost two decades ago, it became clear that the well-defined DNA methylation pattern is essential for the development of organisms. In more recent studies, it has been further unveiled that DNA methylation and its interaction with histone modifications affect the level of gene transcription as well as timing of DNA replication. In consequence, those epigenetic changes influence significant processes within a cell such as proliferation, differentiation, survival, self-renewal, and tumorigenesis. Epigenetic modifications including DNA methylation shape chromatin structure and activity, thus determining the physiological and pathophysiological state of the cell [23,24,25].

During aging and differentiation of the cell, its DNA methylation landscape has to be dynamically modulated. For that to happen not only active DNA methylation but also demethylation has to be possible. DNMT methylated cytosines are transformed to 5-hydroxymethylcytosine (5hmC) by the TET (ten-eleven translocation) family of dioxygenases. Human TET1, a 2-oxoglutarate- and Fe(II)-dependent enzyme, was the first protein identified to catalyze 5mC to 5hmC reaction *in vitro* and *in vivo* [26]. Next, it was shown that not only TET1 but also other TET family proteins, namely TET2 and TET3, are able to convert 5mC to 5hmC [27]. Moreover, Ito et al. have shown that a stepwise process of oxidation of 5mC by TET proteins produces 5hmC, 5-formylcytosine (5fC) and finally 5-carboxylcytosine (5caC) [28]. In embryonic mouse stem cells, 5caC is specifically recognized and excised by thymine-DNA glycosylase (TDG). Thus, transformation of 5mC by TET to its oxidation derivatives 5fC and 5caC, which are further excised by TDG is an example of active DNA demethylation (Figure 2B) [29]. TDG-mediated removal is followed by replacement of the excised residue with an unmodified cytosine by DNA base excision repair (BER) system ([30,31], reviewed in [1]). Demethylation process mediated by TET–TDG–BER may act rapidly and is found as locus-specific demethylation, which might be applied when fast response is required for e.g., environmental changes. A schematic view on TET proteins function and ways of demethylation are shown in Figure 2B. 

The active methylation and demethylation of DNA described above assures tight control of the epigenetic state. However, passive DNA demethylation can also occur under specific circumstances. Inhibition or absence of DNMT1 and UHRF1 or any other dysfunction of DNA maintenance machinery during, e.g., cell division, will result in the passive demethylation (Figure 2A). Replication-dependent loss of 5mC is due to the lack of sufficient maintenance of methylation on the newly formed DNA strand. The replication-dependent dilution of the signal refers only to the loss of 5mC, but not its oxidation derivatives (i.e., 5hmC, 5fC, 5caC) [32].

Finally, DNA methylation acts not only as an epigenetic modification, which affects the chromatin structure and takes part in the regulation of genes transcription, but is also associated with higher mutation rate of methylated DNA sequence. DNA methylation promotes cytosine to thymine transition. It is because 5mC deamination reaction occurs more frequently than deamination of non-methylated cytosine residue [33]. Deamination of 5mC produces thymine, while deamination of unmethylated cytosine produces uracil. Thymine is less likely to be accurately repaired by the DNA repairing system than uracil [34]. In consequence, the initial epigenetic modification (5mC) might be transformed to genetic change.

DNMT proteins actively methylate DNA, TET proteins actively modify 5mC, which is necessary for DNA demethylation. While these two contradictory actions take place in particular loci to control cell development and response to environmental changes, the signal to transfer a methyl group to or from the locus has to be efficiently recognized and read. Methylated DNA is recognized by specific proteins, including the best described methyl-CpG-binding protein 2 (MeCP2). Research taken on MeCP2 established the complexity of its binding to DNA sequence. MeCP2 is well known to interact with mCpG sites, which results in inhibition of genes expression. Interestingly, it was also confirmed that it binds to mCpH sites *in vivo* [9]. More recently, based on *in vitro* experiments, the similar binding affinity of MeCP2 to mCpA and mCpG was confirmed in contrast to mCpT and mCpC, which are bound by MeCP2 to lower extent [35,36]. Not only MeCP2 can read a methylated DNA sequence, many other DNA methylation readers have been reported, specific for different oxidized forms of 5mC and activated in different cell developmental stages in the brain (for review see [37]).

Not only cytosine but also other DNA bases can be methylated. Methylation of adenine (N6-methyladenine, N6-mA) was recently found to be upregulated in glioblastomas (GBM) [38], especially in heterochromatin. Xie et al. suggested that targeting N6-mA regulation might be a good therapeutic strategy to kill GBM stem cells [38]. In the following review, however, we will concentrate on DNA methylation on cytosine residues.

## 2. Introduction to Gliomas

Gliomas constitute ~77% of malignant brain tumors. Due to intrinsic genetic alterations frequent in malignant gliomas, the commonly used drug—temozolomide (TMZ)—is effective only in a small fraction of patients, leaving others only on palliative treatment. World Health Organization (WHO) divided gliomas into four grades based on differentiation status, malignant potential, response to treatment and patient survival rate. Grades I and II are often referred to as low-grade gliomas, while grades III and IV are referred as high-grade gliomas. Despite recent advances in surgery, radiotherapy and chemotherapy, median survival time among patients with grade III tumors is only 2–3 years, while for grade IV glioblastoma (GBM) patients it is only 15 months. GBMs are the most aggressive primary brain tumors and one of the most difficult human malignancies to treat due to multiple dysfunctions of tumor suppressors and oncogenes [39]. Development of new therapy for GBMs is thus the most important task of modern neuroscience and oncology. The Cancer Genome Atlas (TCGA) published datasets for WHO grade II, III and IV gliomas, making it possible to study gene expression, DNA methylation and genetics of low- and high-grade gliomas. In the group of recently added datasets for WHO grade II and III, astrocytic tumors are of main interest, as this histopathologic type is the most common within brain tumors [39,40]. Glioblastomas have been divided into major subtypes: classical, mesenchymal and proneural based on transcriptomic analyses. These subtypes, even though defined by transcriptomic analysis, have been characterized by high frequency of specific somatic alterations, e.g., proneural tumors are enriched in *IDH1* mutations, while classical ones are enriched in EGFR amplification and CDKN2A deletions [41].

In 2016 WHO released a new classification of tumors including gliomas [42]. Due to the rapid development in high throughput methods this new classification of gliomas is mostly based on molecular markers. The main distinction between glioma subtypes in the new classification is based on the *IDH* gene mutation status. As it was shown by other authors and is explained in the following chapters, the *IDH* mutation has a tremendous effect on global DNA methylation pattern.

DNA methylation pattern of the promoter of the *MGMT* (O6-methylguanine–DNA methyltransferase) gene was shown to be a prognostic marker for GBM patients treated with the TMZ [43]. Patients with silenced *MGMT* gene expression due to high gene promoter methylation were found to have a favorable outcome, when compared to patients with an unmethylated *MGMT* promoter. In fact, in patients without *MGMT* methylation, TMZ treatment did not give any benefit as compared to the radiotherapy alone [43]. Last year, glioma-specific DNA methylation pattern was detected in cell-free circulating tumor DNA obtained from glioma patients [44]. Recently, it was suggested that glioma detection and monitoring based on blood-derived DNA methylation will soon be possible [45]. This underlines the fact, that DNA methylation patterns in gliomas are very important and need to be very well understood.

## 3. Derivatives of Methylcytosine

It is still a matter of debate, whether 5mC derivatives represent only intermediate states in methylation–demethylation machinery or serve themselves as important epigenetic marks involved in the regulatory systems. High levels of 5hmC were found to be present in embryonic stem cells (ESCs) and Purkinje neurons [26]. The high abundance of 5hmC was confirmed to participate in methylation-mediated gene silencing, and even a single 5mC oxidation to 5hmC reduced the binding affinity of methyl-CpG-binding protein MeCP2 to DNA by at least an order of magnitude [46]. Contrariwise, Yildirim et al. have suggested that methyl-CpG-binding domain protein 3 (MBD3) binds with a high affinity to regulatory sequences, which are enriched for 5hmC epigenetic mark [47]. MBD stands for “methyl-CpG-binding domain” due to homology of MBD3 to MeCP2. MBD3 preferentially binds to 5hmC enriched probes in comparison to 5mC probes. Since MBD3 co-localizes with TET1 and 5hmC, Yildirim et al. have proposed a model in which TET1 transforms 5mC to 5hmC to recruit MBD3 [47]. Moreover, it has been revealed that MBD3 plays a role in the regulation of bivalent genes in ESCs. Similarly to TET1, MBD3 is present in CpG-rich promoters of genes that are bound by Polycomb and was found to be essential for their normal expression [47].

More recently, 5hmc together with MeCP2 have been shown to constitute a cell-specific epigenetic mechanism for regulation of chromatin structure and gene expression. MeCP2 was identified as the major 5hmC-binding protein in the brain and it was demonstrated that its binding affinity to 5hmC- and 5mC-enriched DNA regions is similar [48], pattern surprisingly different from that observed in ESCs [47]. Further studies indicated that MeCP2 binding affinity to 5hmC is also context dependent, determined by the nucleotide next to 5hmC [35,49]. Hydroxy-methylation (5hmC) of DNA, even though classically described as an intermediate step of DNA demethylation, can have also a paradoxical effect on DNA methylation. Due to the weak binding affinity of DNMT1 to 5hmC, DNA methylation cannot be executed. Not only 5hmC but also 5fC and 5caC were confirmed to recruit specific sets of proteins in a cell-type-dependent manner. Readers of 5mC derivatives seem to show high specificity for a certain type of oxidized forms of 5mC [37,50].

Since TET family of enzymes plays a central role in the conversion of 5mC to 5hmC and to other derivatives of 5mC, more attention has been paid to study the interplay of 5hmC and TET enzymes in various biological pathways [51,52], as well as in cancer development [53]. It is especially important in the context of gliomas, because 5hmC displays the highest levels in the brain ranging from 0.4% to 0.7% of the total cytosine content in comparison to other tissues, e.g., lung or liver, where it appears at the levels below 0.2% [54,55,56], and in glioblastoma cells it reaches about 1% [57].

All three TETs (TET1, TET2 and TET3) are expressed in the brain and they seem to be associated with neuronal differentiation and neural progenitor cell formation [58,59]. It is known that TET family proteins can be inhibited by 2-hydroxyglutarate (2HG) [60], an oncometabolite synthetized by cells carrying specific mutations. The 2HG oncometabolite is generated as the outcome of mutations in isocitrate dehydrogenase 1 and 2 genes (*IDH1* and *IDH2*) and is produced apart from a physiological product of IDH enzymatic activity: α-ketoglutarate [61]. The *IDH* mutation status is a well-known significant molecular prognostic marker of glioma patients as well as classification feature of glioma subtypes in the World Health Organization categorization [42]. *IDH1* and *IDH2* are mutated in over 75% of low grade gliomas and secondary glioblastoma multiforme (GBM) [62,63]. 2HG causes the depletion of 5hmC in cancers by promoting hypermethylation of specific loci [64,65]. Methylated DNA sites cannot be actively demethylated by TET, because of 2HG accumulation. A schematic view on the alteration of TET function by 2HG is shown in Figure 1D. There are clear pieces of evidence that inhibition of TET-mediated oxidation of 5mC to 5hmC occurs not only in DNA but also in RNA [66].

Recently it was shown that TET1 upregulation might be responsible for the elevated levels of 5hmC in proneural glioma subtypes, in case when none of the tumor samples represented *IDH* mutant status [57]. Takai et al. have confirmed experimentally that TET1 is required for glioma cells proliferation. Its knockdown inhibited glioblastoma progression resulting in longer survival of mice, but with TET1 becoming overexpressed again, the tumorigenicity was restored. Moreover, TET1-catalyzed enrichment of 5hmC is required for overexpression of genes participating in cancer-related pathways and neuronal functions. The TET1 knockdown introduced in mice resulted in decreased expression of the *EGFR*, *AKT3*, *CDK6* and *BRAF* genes [57]. The genes can become overexpressed again when TET1 expression is restored.

## 4. Glioma Specific DNA Methylation Patterns

### 4.1. G-CIMP Phenotype

In 1999 Toyota et al. discovered a pattern of hypermethylated CpG islands within promoter regions of the tumor suppressor genes in colorectal cancer [67]. The discovered pattern was named a CpG island methylator phenotype (CIMP). About a decade later a similar pattern was also described in gliomas and named a glioma-CpG Island Methylator Phenotype (G-CIMP) [68]. The G-CIMP phenotype occurs most frequently in gliomas of grade II, III and secondary glioblastomas [68]. As it was earlier mentioned, 75% of gliomas of grade II/III and secondary glioblastomas are *IDH*-mutated. It is therefore safe to state that *IDH* mutations contribute to the G-CIMP phenotype [68]. Patients manifesting the G-CIMP phenotype, especially those harboring also a 1p19q co-deletion have better prognosis than patients not manifesting this phenotype. The lack of G-CIMP phenotype, occurring in most of glioblastomas, does not mean that there are no aberrations in DNA methylation patterns. Alterations of DNA methylation in GBMs are more difficult to summarize in one concise phenotype, or it is still a grey area of research.

The characterization of G-CIMP phenotype concentrated on the pattern of 5mC levels, ignoring 5hmC levels since 450k Illumina arrays technology in its original design could not distinguish between 5mC and 5hmC. Lately, 5hmC was also shown to have a specific pattern in gliomas. Thanks to genome-wide map of 5hmC in human ESCs, it has been shown that 5hmC is frequently present in enhancers, specifically active enhancers (i.e., enhancers enriched with H3K4me1 and H3K27ac marks) as well as in gene bodies, implicating its role in the regulation of gene expression [69]. More recently, the aberrant DNA hypermethylation (5mC) in glioma within CpG island shores was found to be 5hmC-dependent. Interestingly, shores depleted in 5hmC became 5mC hypermethylated and were found to be enriched in H3K4me2 presenting novel chromatin signature [70].

### 4.2. IDH-Related Phenotype

Turcan et al. have performed functional studies of the effect of *IDH* mutation on global DNA methylation [71]. Authors have introduced mutant *IDH* or *IDH* wild type into immortalized human astrocytes, using retroviral infection. They have observed more than 44 thousands of differentially methylated CpG sites with more than 30 thousands being hypermethylated in *IDH*-mutant astrocytes. Interestingly, hypermethylated sites were highly enriched for Polycomb Repressive Complex 2 (PRC2) targeted loci. Moreover, Turcan et al. have found a significant decrease of 5hmC levels in *IDH*-mutant astrocytes [71]. Since 5hmC is an intermediate in TET-dependent step of demethylation, TET inhibition results in the accumulation of 5hmC. 

Later the same scientific group has shown that the phenotype attributed to *IDH* mutation is developing for a long time. Immortalized human astrocytes presented such a phenotype only after 30 passages [72]. Further, in the experimental design the *IDH* mutant expression was switched off for 40 passages to observe whether IDH-dependent methylation profile is reversible and if it is, to what extent. Only up to 75% of IDH-dependent DNA methylation loci got back to the initial level of methylation after 40 passages of *IDH*-mutant being switched off. Turcan et al. suggest that the remaining 25% of loci that maintain their aberrant methylation level can be considered as irreversible changes caused by *IDH* mutation [72]. While it may be the case, it should be kept in mind, that the experiment was done on immortalized astrocytes, to which *IDH*-mutant construct was introduced by transfection. This model cannot be treated as a physiological model of *IDH* mutation. It is also of note that the experiment of *IDH*-mutant being switched off was maintained for 40 passages, and we do not know if it was long enough to reverse the effect of long-term exposure to 2HG. The effect of 2HG on DNA methylation accumulates in time and reversion of its action may just take longer than it had been designed in the study.

## 5. DNA Methylation and Chromatin Modifications

### 5.1. Adult High-Grade Gliomas

One of the first described links between chromatin modifications and DNA methylation was a discovery of a dual function of protein from the Polycomb group, EZH2 (Enhancer of Zeste homolog 2). It was shown that apart from its canonical role of a histone methyltransferase, EZH2 also controls DNA methylation [73]. Vire et al. have showed that polycomb repressive complex (PRC) can physically interact via EZH2 with DNA methyltransferases. EZH2 recruits DNA methyltransferases to target sequences to place repressive marks on DNA [73]. This interesting mechanism can explain great overlap of repressive histone modifications and DNA hypermethylation in promoters. Histone repressors and DNA methyltransferase can be guided by the same protein complex.

Another important mechanism that associates chromatin modifications with DNA methylation is directly related to 2HG production in *IDH1/2* mutated gliomas. The effect of this metabolite on DNA methylation was already described in Section 3. Surprisingly, the same metabolite was shown to directly affect histone lysine demethylases. In one of early studies it was shown that 2HG might inhibit a range of human 2-Oxoglutarate (2OG) oxygenases, including JmjC domain-containing lysine demethylases. One of the most potent effects was observed for N-methyl lysine demethylase KDM4A (formerly known as JMJD2A) [74]. KDM4A histone demethylase family is believed to be involved in the demethylation of H3K36me3 and H3K9me3 lysine sites [75]. Inhibition of KDM4A by 2HG may lead to histone hypermethylation, which in turn causes repression of gene expression.

Another study showed that 2HG may affect also other demethylases, such as KDM2A, and influence a wide range of lysine histone methylation positions, including H3K4, H3K9, H3K27 and H3K79 [60]. Inhibition of histone demethylase may have a synergistic effect with 2HG-driven blocking of TET enzymes activity. Both types of alterations of epigenetic marks on DNA and histones in tumor cells may repress genes that are activated in the process of cell differentiation.

**Figure 3:**
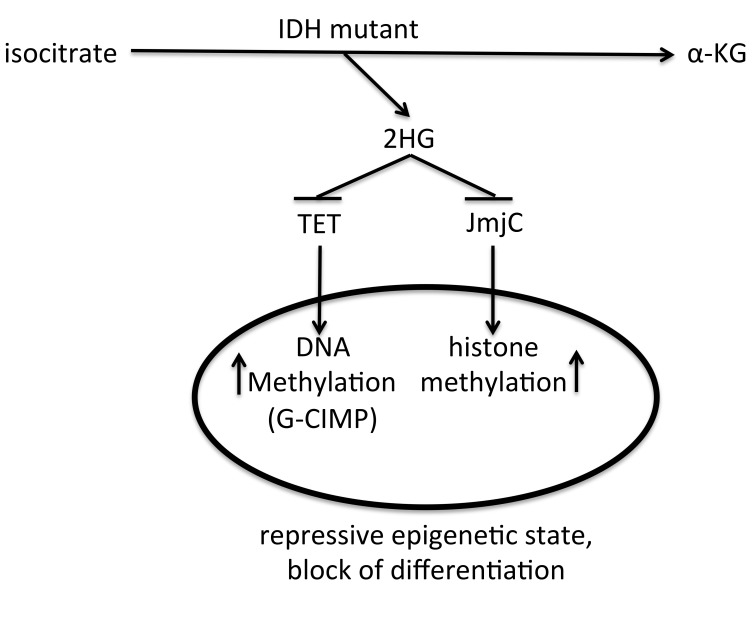
Schematic view on the role of IDH mutant on the epigenetic state of a cell. IDH mutant produces 2HG, that inhibits TET DNA demethylases and JmjC histone demethylases, causing increased DNA and histone methylation levels, which lead to a repressive epigenetic state and block of differentiation.

In a more recent report, it has been shown that in the cells with ectopically expressed mutant *IDH1* or *IDH2*, there is a statistically significant increase in repressive histone marks, namely H3K9me3 and H3K27me3, but not of activating marks, such as H3K4me3 [76]. DNA methylation is a more stable epigenetic modification in comparison to histone marks, which are believed to be more plastic. In fact, in the model of ectopic mutant *IDH1* expression, the changes in histone marks were observed five cell passages before any alterations in DNA methylation were observed [76]. It is important to note, that the block of differentiation caused by 2HG oncometabolite, produced by the gain-of-function mutation in *IDH 1/2* is primarily executed by histone methylation changes, followed by more permanent DNA methylation changes. A schematic view of the effect of *IDH 1/2* mutations on epigenetic landscape is shown in Figure 3.

Another interesting mechanism of interplay between DNA methylation and histone modifications is related to the chromatin target of PRMT1 (CHTOP) methylosome complex. In contrast to other cancers, 5hmC levels in proneural subtype of glioblastoma were found to be increased, most likely due to high expression and activity of TET1 enzyme [57]. TET1 enzyme, as it was described earlier, catalyzes a transition of 5mC to 5hmC. The question arises, how these high levels of 5hmC levels are maintained, since 5hmC is a necessary intermediate in the process of DNA demethylation and was believed to be promptly oxidated. Takai et al. showed that CHTOP is recruited to sites that are 5hmC-methylated and methylates H4R3, what seems to activate cancer-related genes but also protects 5hmC from oxidation to 5fC and 5caC [57]. CHTOP knockdown results in a global drop in the 5hmC levels and CHTOP rescue restores elevated levels of 5hmC. CHTOP, by putting H4R3 histone marks, stabilizes 5hmC. Knockdown of CHTOP inhibits tumorigenicity of glioblastoma cells resulting in longer mice survival, pointing to the importance of 5hmC levels for the prognosis of patients with proneural glioblastoma tumors [57].

### 5.2. Pediatric High-Grade Gliomas

In pediatric high-grade gliomas, epigenetic changes are also extremely important in oncogenesis, but the pattern of epigenetic deregulation is vastly different. While in adult high-grade gliomas, histone and DNA epigenetic changes are believed to maintain more repressive and undifferentiated state of the genome, it seems that some pediatric gliomas are driven by DNA and H3K27me3 hypomethylation [77,78]. As H3K27me3 is a known repressive mark, hypomethylation of this histone residue will cause chromatin de-repression. Histone genes can be also mutated. Histone variant H3.3 mutations are found in around 50% of pediatric high-grade gliomas affecting positions K27 and G34, thus leading to lysine to methionine (K27M) substitution and glycine to arginine or valine (G34R or G34V) substitutions, respectively [77,78]. Interestingly, tumors with the K27M substitution may also carry a PRC2 dysfunction. As it was described before, PRC2 is a repressive complex, which maintains a repressive state of chromatin. Thus, K27 mutants, affecting the function of PRC2 complex will lead to changes in both DNA and histone methylation. G34R histone mutation was recently described to be affecting KDM4 family of histone demethylases. Namely, KDM4 demethylates histones at H3K9 and H3K36 positions. By the dysfunction of KDM4A, G34R mutant affects the level of H3K9 and H3K36 histone methylation [79]. Not much is known about the mechanism of how the G34 variant may affect DNA methylation.

## 6. Transcription Factors Binding Affected by DNA Methylation 

In the recent report of Yin et al., many transcription factors were identified as being sensitive to cytosine methylation [80]. Using a method called bisulfite SELEX (systematic evolution of ligands by exponential enrichment) those authors have confirmed and identified new cases of differential binding of many transcription factors depending on cytosine methylation status within transcription factor binding sites. Indeed, CpG methylation has a major effect on transcription factors (TFs) binding to DNA and, surprisingly, it can have both a promoting or inhibitory effect on the binding to DNA (Figure 1C). Certain TF families, like homeodomain, POU and NFAT (nuclear factor of activated T-cells) prefer DNA-methylated sites, while bHLH (basic Helix-Loop-Helix), bZIP (basic Leucine Zipper Domain) and ETS (E-twenty-six) prefer unmethylated sites [80]. Information about the effect of DNA methylation on TF binding was recently used in a methylation-sensitive database of TF binding motifs [81]. An interesting subset of homeodomain TFs are *HOX* genes, that are believed to be sensitive to DNA methylation occurring in their binding motifs [80]. What has also been shown in glioblastoma, is a cluster of *HOX* genes, differentially methylated in short-term and long-term glioblastoma survivors [82], associated with stem-cell signature [83]. SOX2 ((Sex Determining Region Y)-box 2), that is also considered to be a stem cell-related TF, has a hypomethylated promoter in glioma, when compared to normal cell lines [84]. The SOX family of transcription factors seems to be important in glioma progression. As recently shown, the DNA methylation pattern of recurrent glioma tumors defined as G-CIMP-low is mainly enriched in enhancers with AP-1/SOX binding elements [85].

DNA methylation affects yet another key player in neural stem cells, namely the REST transcription factor [86]. REST stands for RE1-silencing transcription factor and was until recently also called NRSF—neuron-restrictive silencer factor. A canonical function of this transcription factor is to control the neuron differentiation process [87]. REST and its corepressor are believed to repress neuronal gene expression in non-neural terminally differentiated cells, as well as was proven to be important in shaping neuronal plasticity in the developing brain [87]. REST regulates a transition from stem/progenitor cells and plays a crucial role in a physiological processes in the brain, promoting transcription of genes from neuronal lineage, but also repressing a number of genes [87]. REST cannot act alone and it was found to recruit many epigenetic factors that may repress or activate gene expression by imprinting active or repressive marks on histones and DNA. REST can bind directly to its own motifs or can be recruited by MeCP2 (methyl-CpG binding protein 2) [87]. It was shown recently, that DNA methylation may affect REST binding in human glioma and that its binding to specific cytosines may be prognostic for patients survival [88]. The effect of REST binding may go beyond simple TF-gene regulation scenario, since REST was shown to recruit many histone modifiers: 1) HDAC1/2—histone deacetylases known to be recruited as a part of both coREST and NcoR repressive complexes [87,89]; 2) KDM1A (LSD1)—a flavin dependent lysine 4 of histone protein H3 (H3-Lys4) demethylase recruited as a part of LSD1-CoREST/nucleosome complex [89], which removes methyl groups from histone 3 mono- or di-methylated at lysine 4 (H3K4me1, HSK4me2) [90,91]; 3) EHMT2—euchromatic histone lysine methyltransferase 2 (also known as site-specific histone methyltransferase G9a), which promotes dimethylation of histone 3 at lysine 9 (H3K9me2) [87,92,93]; 4) SUV39H1—suppressor of variegation 3–9 homolog 1, a histone methyltransferase that trimethylates lysine 9 of histone H3, which results in transcriptional gene silencing [94]. Moreover, REST may indirectly affect DNA methylation in neurons by recruiting TET3 and inducing hydroxymethylation (5hmC) and subsequent gene expression activation.

Although there have been already several reports describing transcription factors, which are affected by DNA methylation in gliomas, including SOX, HOX and REST, we are still far away from having a complete view on the sequence of events that lead to deregulation of transcription factor pathway networks in the context of altered DNA methylation leading to glioma progression. One of the best-known examples of the influence of DNA methylation on the transcription factor binding affinity that has tremendous effects on the development of IDH mutation related phenotype is described in the next paragraph.

## 7. DNA Methylation Affecting Chromatin Contacts

Another very important transcription factor that was shown to be affected by DNA methylation is CTCF (CCCTC-binding factor). An alteration in global CTCF binding strongly affects gene expression patterns, mostly by disturbing the 3D chromatin structure. It is mainly attributed to the fact that CTCF is not a typical transcription factor. It was shown, that CTCF is crucial for creating chromatin loops and boundaries between separate chromatin compartments [95,96]. CTCF can be defined as a string wrapper, that is important to connect promoter to its enhancer when necessary and put insulator to promoter when it is required. Disruption of this mechanism may have tremendous consequences, since in physiological conditions in differentiating cells, as well as in terminally differentiated cells, specific genes should be silenced while others kept active at a specific time and/or tissue location. CTCF binding can be also affected by DNA hypermethylation related to *IDH* mutations. Upon global hypermethylation of CTCF binding sites in *IDH*-mutated gliomas a massive disruption of boundary elements occurs that completely changes the topological organization of chromatin [97]. Flavahan et al. described an example of *PDGFRA* (platelet derived growth factor receptor alpha) gene, which is activated by the *FIP1L1* (factor interacting with PAPOLA and CPSF1) gene enhancer even though they are separated by a 900kB distance, and are kept apart by CTCF [97]. In the case of hypermethylation of this CTCF site, *PDGFRA* binds to the *FIP1L1* enhancer. Binding of the *PDGFRA* promoter to the *FIP1L1* enhancer increases expression of the *PDGFRA* gene. A schematic view on how the change in DNA methylation may affect CTCF related gene expression regulation is shown in Figure 1F. Another example was shown in neural stem cells model with an introduced *IDH1* mutation and subsequent *TP53* (tumor protein p53) and *ATRX* (Alpha Thalassemia/Mental Retardation Syndrome X-Linked) knockdowns, which was supposed to mimic a potential way of oncogenesis of lower grades gliomas of astrocytic origin [98]. Changes of methylation in *IDH1/TP53/ATRX*-triple mutant neural cells affected CTCF binding sites around the *SOX2* gene and consequently a local chromatin structure. This, in turn, resulted in blocking of normal NSCs (neuronal stem cells) differentiation. Strikingly, switching on one transcription factor at a wrong time causes neural stem cells to enter an oncogenic path. In this way, the study suggests that restoring *SOX2* expression may be sufficient to rescue neural stem cells differentiation [98].

Large fragments of DNA, that are methylated, are usually localized within the condensed part of the chromatin (heterochromatin). One of the important chromatin chaperones is ATRX, which is frequently mutated in gliomas [39]. It has been shown, that *ATRX* loss in glioma grade II and III tumors, is an alternative way of telomere elongation [99], alternative to telomerase reverse transcriptase (*TERT*) promoter mutations [100]. Loss of *ATRX* was also shown to affect the level of methylation at the chromosome ends, where telomeric regions are located [100]. A schematic view on how ATRX may interact with methylated DNA is shown in Figure 1E.

## 8. Conclusions

In a nutshell, gliomas are characterized by global changes in DNA methylation. They are most frequently triggered by *IDH* mutation. The *IDH* mutation not only changes global DNA methylation but also alters histone modifications. G-CIMP gliomas associated with *IDH* mutation status exhibit hypermethylated phenotype, causing a repressive epigenetic state and block of differentiation. Changes in DNA methylation in gliomas have been shown to alter the binding affinity of several transcription factors, including SOX and HOX, as well as the REST transcription factors. One of the most interesting examples is CTCF, which was described as an insulator, important for the maintenance of chromatin structure. DNA methylation changes within CTCF binding sites lead to global alterations of chromatin structure. Loss of *ATRX* leads to changed DNA methylation pattern at the ends of chromosomes. In pediatric gliomas, changes in histone genes are observed, causing dysfunction of PRC2 complex, which leads to alteration in both histone and DNA epigenetic landscape.

## 9. Future Perspective

Global DNA methylation in gliomas may soon become a diagnostic and prognostic marker. As changes in DNA methylation are so well described, we can expect some new therapeutic interventions targeting IDH- or G-CMIP-related phenotypes. With the advent of 3rd generation sequencing it may be soon possible to read long DNA sequence together with its modifications, including DNA methylation. This will be the next step, to link DNA methylation on a single molecule level with DNA alterations and to acquire a single molecule, and a single strand DNA methylation resolution. Moreover, DNA methylation pattern detection in cell-free circulating tumor DNA may soon become an early diagnostic and monitoring tool.
